# Short-term effects of thinning on the growth and soil improvement of typical stands in the Yellow River Delta

**DOI:** 10.3389/fmicb.2025.1585176

**Published:** 2025-05-07

**Authors:** Wenjing Liu, Lichao Wang, Jiangbao Xia, Yue Lu, Xianguo Zong

**Affiliations:** ^1^Shandong Key Laboratory of Eco-Environmental Science for the Yellow River Delta, Shandong University of Aeronautics, Binzhou, China; ^2^Binzhou Hydrographic Bureau, Binzhou, China

**Keywords:** thinning, stand density, stand growth, soil physical and chemical properties, Yellow River Delta

## Abstract

The effects of thinning were studied on stand growth and the physicochemical soil characteristics of typical plantations in the Yellow River flood plain in northern Shandong Province. Growth indices and soil physical and chemical indices were monitored and in 8-year-old plantations of *Fraxinus chinensis*, *Salix matsudana*, and *Ulmus pumila* in the Yellow River Delta. Data were collected at an initial stand density of 3 m × 3 m and 3 years after artificial thinning to a stand density of 3 m × 6 m. (1) Thinning promoted growth, and the effect on the *U. pumila* plantation was the greatest. The diameter at breast height, tree height and crown width increased by 41.28, 19.98, and 59.49%, respectively. (2) Thinning increased the soil moisture at the studied plantations, reduced the soil bulk density, and increased the soil porosity and the water holding capacity. The results differed among the plantations, with the greatest improvement occurring at the *U. pumila* plantation. (3) Thinning had a greater effect on inorganic than on organic soil nutrients according to forest type; the changes in nitrogen, phosphorus and potassium contents in different soil layers substantially varied with the species planted. Most variables significantly differed at the *S. matsudana* plantation, and the changes in different soil layers were inconsistent. (4) Thinning significantly increased the correlations between stand growth and soil physicochemical properties. Thinning positively affected forest growth and soil physicochemical properties, but the variation in each index across the different plantations was quite different. Thus, thinning could help promote the stable and sustainable development of forest plantations in the Yellow River Delta, and these results provide a reference for the rational management of plantations.

## 1 Introduction

Soil erosion and water loss have caused long-term damage to the Yellow River flood plain in China (Du et al., 2016). Since 2000, the area has gradually shifted to experiencing light erosion due to the implementation of artificial shelter forests (Fu et al., 2021). Although artificial shelterbelts can provide critical services related to windbreaks, sand fixation, and soil and water conservation, the initial planting density of most artificial shelterbelts does not conform to the principle of “suitable for trees,” resulting in poor tree growth and affecting the comprehensive benefits of shelterbelts (Liu et al., 2019). Thinning is a key forest management method. Thinning provides increased light, space and nutrition for retained trees by reducing tree density, promotes rapid tree growth, and helps develop a reasonable stand structure with respect to tree species, age and spatial distribution, thereby improving the stability and resistance to interference to stand interference. Thinning indirectly and directly affects the symbiosis between plant roots and soil microorganisms and promotes nutrient cycling in the community (Palta et al., 2024). Research on the effects of thinning on plantations has focused on the functional and phylogenetic diversity of regenerated woody plants, species composition (Xie et al., 2023), abundance and diversity of deadwood in plantations (Li M. et al., 2023), soil properties (Zhang et al., 2023), microbial functions and diversity (Xu et al., 2020), vegetation diversity (Wang G. R. et al., 2021; Sun et al., 2020), forest biomass (Baul et al., 2020), improvement in plant drought resistance (Manrique-Alba et al., 2020), decomposition of forest litter and soil fertility (Perez-Alavez et al., 2021; Zhou et al., 2021; Gong et al., 2020), and forest carbon storage (Tao et al., 2019; Jörgensen et al., 2021); however, studies of how thinning affects stand growth characteristics in the windblown sandy area of the Yellow River Delta Plain are relatively rare, and the relationships among these factors are unclear. Elucidating the relationships between thinning and stand growth characteristics could provide guidance for implementing more effective and sustainable forest management strategies at plantations.

Thinning changes the forest base area (de Oliveira et al., 2021), water use efficiency (Ma et al., 2020), photosynthesis rate (Niccoli et al., 2020; Chi et al., 2020), and soil quality (Romeo et al., 2020). Therefore, the life activities of soil microorganisms and plant root systems, are affected, which in turn influence the physical and chemical properties of the soil (Palta et al., 2024). Changes in soil physical and chemical properties are direct responses to changes in stand density, which indirectly affect the stability of an ecosystem and are important in soil conservation (Yamagishi et al., 2022; Qu et al., 2021). Therefore, it is essential to study the effects of thinning on soil improvement during plant growth. Studying of soil physical and chemical properties (such as bulk density, porosity, and nutrient content) and microbial characteristics before and after thinning is helpful for understanding the different effects of thinning (Zhang et al., 2023). However, few studies have investigated the effects of thinning on the physical and chemical properties of soils in the Yellow River Delta Plain.

Three 8-year-old plantations of *Fraxinus chinensis*, *Salix matsudana*, and *Ulmus pumila*, which were selected as the research objects, are located at the Wangzhuang forest farm in the Yellow River Delta Shandong Province and had identical initial site conditions. Our aim was to investigate the impact of artificial thinning on growth and soil physical and chemical properties. Specifically, we examined the effects of reducing the initial stand density from 3 m × 3 m to 3 m × 6 m. We focused on the Yellow River floodplain area and that stand density regulation significantly impacts forest growth and soil properties.

## 2 Materials and methods

### 2.1 Overview of the study area

The study area is at Wangzhuang Forest Farm, Lijin County, Dongying city, Shandong Province (118°16′01″E, 37°34′08″N), which is in the North China Depression; this area has a warm temperate semihumid monsoon climate with an elevation of 8–9 m, an annual average temperature of 13.3°C, a maximum temperature of 39.9°C, and a minimum temperature of −20.2°C. The annual sunshine duration is 2834.7 h, the annual rainfall amount is 526.2 mm, the frost-free period averages 215 days, and the year is divided into four distinct seasons. The soil is a fluvo-aquic soil with a sandy texture, a structural composition of sand:silt:clay = 60%:30%:10%, a salt content of <1.5‰, and a pH of 7.5–8.0. The major afforestation tree species are *F. chinensis*, *U. pumila*, and *S. matsudana*. The major herbaceous plants in woodlands in the area are *Tribulus terrestris*, *Pennisetum alopecuroides*, *Agriophyllum squarrosum*, and *Artemisia lavandulifolia*.

### 2.2 Sample setting and survey method

#### 2.2.1 Sample setting

In January 2020, the *F. chinensis*, *U. pumila*, and *S. matsudana* plantations that had been growing for 8 years in the area were thinned by half. This involved artificially thinning the initial row spacing from 3 m × 3 m to 3 m × 6 m. The initial *F. chinensis*, *U. pumila*, and *S. matsudana* plantations each had three plots of 20 m × 20 m, with 18 plots in total. To collect initial density data, a stand growth survey and soil sample collection were performed in January 2020. Stand growth data and soil samples representative of artificial thinning were collected in September 2023, 3 years and 9 months after thinning. The relevant indicators were then measured.

#### 2.2.2 Plantation tree measurements and ratio analysis

Three plots were established in two distinct areas representative of different forest densities, where each area contained different forest types and every tree was examined. The height, diameter at breast height (DBH), and crown width of each tree were measured via Bruce altimetry, a DBH ruler, and a tape measure. The breast-height shape coefficient, height-diameter ratio, crown-height ratio, crown-diameter ratio, and stem width were calculated. The breast-height shape coefficient is the ratio of the trunk volume to the volume of the corresponding cylinder, with the DBH section as the cross section, which indicates the volume of the trunk.


$$\mathrm{Breast height shape number}=V/\left(g_{1.3}\times H\right)=\frac{\pi }{4}{D_{1.3}}^{2}\times H$$

*V*, stem volume; *g*_1.3_, section area at breast height; *D*_1.3_, diameter at breast height; and *H*, tree height.

Tree height is an important basis for assessing site quality and forest growth status and dividing forest layers (Chen et al., 2020). DBH is a common index used to measure the thickness of trees and is directly related to the volume and biomass of trees (Bhandari et al., 2021). Crown width refers to the distance between the trunk of the tree and the widest part of the crown. It is an important index for measuring the growth status and stand structure of trees (Peng et al., 2022). Indices such as DBH, height-diameter ratio, crown-height ratio and crown-diameter ratio can provide a quantitative description of tree morphology and growth status, which is helpful for evaluating the growth rate, morphological characteristics and age of trees (Negishi et al., 2020). Stem fullness is an index used to measure the shape of a tree trunk and reflects the symmetry of the tree trunk and the uniformity of growth. Stem fullness may also be related to the physiological health of trees and their ability to adapt to environmental stress.

### 2.3 Sample collection and determination methods

#### 2.3.1 Soil sample collection

*F. chinensis* from the initial area (WoT) is used here as an example to describe the method of soil sample collection. Three 5 m × 5 m areas were randomly selected at the initial *F. chinensis* plantation, and five sampling points were selected in each 5 m × 5 m area via the “S” sampling method. Soil samples were collected at depths of 0–20, 20–40, and 40–60 cm. The samples of the same soil layer obtained at 5 sampling points were uniformly mixed into one sample per region. The same sampling method was used for *U. pumila* and *S. matsudana* in the initial area (WoT) and for *F. chinensis*, *U. pumila*, and *S. matsudana* in the thinned area (WT).

#### 2.3.2 Determination methods

The following soil physical and chemical indices were measured via the ring knife immersion method (Zhang et al., 2021): soil bulk density (g/cm^3^), soil porosity (%), and soil water holding capacity (%) (Gao et al., 2022). Before the soil samples were taken, the mass of the ring knife was recorded as *W*_0_, and the volume was recorded as *V*; after each sample was taken, the total mass of the ring knife and the soil was recorded as *W*_1_. The ring knife and soil samples were immersed in ultrapure water for at least 12 h so that the water level line did not exceed the upper edge of the ring knife. When the soil sample was completely saturated, the mass of the ring knife and the soil mass was recorded as *W*_2_. Finally, the soil and the ring knife were placed in the oven and dried to a constant weight for at least 12 h, and the final weights of the ring knife and the soil were recorded as *W*_3_.


$$\mathrm{Bulk density}\;\left(g/{\mathrm{cm}}^{3}\right)=\left(W_{3}-W_{0}\right)/V$$

$$\mathrm{Total porosity}=\left(W_{3}-W_{2}\right)/V\times 100\%$$

$$\mathrm{Capillary porosity}=\left(W_{3}-W_{1}\right)/V\times 100\%$$

Noncapillary porosity=Total porosity−capillary porosity

Soil total nitrogen (TN, mg/kg) was measured via the semimicro-Kjeldahl method (Cui et al., 2021). The remaining parameters were determined as follows: soil ammonia nitrogen (AN, mg/kg) (Cui et al., 2021), 2 mol L^−1^ KCl extraction-distillation; soil nitrate nitrogen (NN, mg/kg) (Cui et al., 2021), phenol disulfonic acid colorimetry method; soil total phosphorus (TP, mg/kg) (Qu et al., 2021), NaOH melting-Mo-Sb colorimetry; soil available phosphorus (AP, mg/kg) (Qu et al., 2021), 0.5 mol L^−1^ NaHCO_3_ method; soil total potassium (TK, mg/kg) (Cui et al., 2021), NaOH melting-flame photometry; soil available potassium (AK, mg/kg) (Cui et al., 2021), NH_4_OAC extraction and ESA flame photometry; and soil organic matter (OC, g/kg) (Yang et al., 2022), potassium dichromate volumetric method with external heating.

### 2.4 Data processing method

Excel 2016 and SPSS 26 software were used for data processing to perform one-way ANOVA on the final dimensions (DBH, tree height and crown width) of the stands and on the tested soil physical and chemical parameters of the tested soils. The Duncan method was used to compare the significant and nonindigenous differences between the indices. Pearson correlation was used to the correlations between indices. The entropy weight method was used to calculate the weight of each index.

## 3 Results

### 3.1 Stand growth characteristics before and after thinning

#### 3.1.1 Morphological characteristics of trees

Table 1 shows that after the stand density decreased from 3 m × 3 m to 3 m × 6 m, the rates of growth in the DBH, tree height and crown width at the *U. pumila* plantation increased significantly (*p* < 0.05), and the final average dimensions were 41.3, 20.0, and 59.5%, higher, respectively. The rate of growth of the morphological characteristics of the *F. chinensis* and *S. matsudana* plantations did not change significantly due to the decrease in stand density.

**Table 1:** Tree morphological characteristics of different plantations before and after thinning.

Morphological characteristics	Plantations					
	*Fraxinus chinensis*		*Salix matsudana*		*Ulmus pumila*	
	WoT	WT	WoT	WT	WoT	WT
D (cm)	13.3 ± 2.46^a^	14.4 ± 1.64^a^	14.9 ± 1.00^a^	15.0 ± 2.19^a^	11.7 ± 2.22^b^	16.5 ± 2.83^a^
H (m)	9.1 ± 0.45^a^	9.5 ± 0.97^a^	8.9 ± 0.32^a^	9.0 ± 0.45^a^	10.5 ± 0.81^b^	12.6 ± 0.99^a^
C (m)	4.0 ± 0.67^a^	4.5 ± 0.43^a^	2.5 ± 0.43^a^	2.8 ± 0.44^a^	3.2 ± 0.67^b^	5.0 ± 0.72^a^
Bhff	0.55 ± 0.01^a^	0.54 ± 0.02^a^	0.54 ± 0.02^a^	0.54 ± 0.01^a^	0.56 ± 0.01^a^	0.51 ± 0.01^b^
Rhd (m cm^−1^)	0.73 ± 0.04^a^	0.64 ± 0.03^a^	0.60 ± 0.02^a^	0.60 ± 0.03^a^	0.92 ± 0.05^a^	0.78 ± 0.03^b^
Chr (m m^−1^)	0.27 ± 0.02^b^	0.49 ± 0.01^a^	0.43 ± 0.02^a^	0.40 ± 0.02^a^	0.37 ± 0.01^a^	0.31 ± 0.02^b^
Rcd (m cm^−1^)	0.31 ± 0.01^a^	0.31 ± 0.01^a^	0.19 ± 0.01^a^	0.16 ± 0.01^a^	0.28 ± 0.03^a^	0.31 ± 0.01^a^
Sf	1.06 ± 0.01^a^	1.09 ± 0.03^a^	1.05 ± 0.01^a^	1.08 ± 0.05^a^	1.07 ± 0.02^a^	1.05 ± 0.02^a^

The data are the mean ± standard deviation; different lowercase letters in the same column indicate significant differences after the same stand density reduction (*p* < 0.05); H, tree height; C, crown width; D, diameter at breast height; Bhff, breast height form factor; Rhd, ratio of height to diameter; Chr, crown-height ratio; Rcd, ratio of crown to diameter; Sf, stem fullness; WoT, without thinning; WT, with thinning.

#### 3.1.2 Effects of thinning on the stand morphological characteristics of plantations

Table 1 shows that after the decrease in stand density, the breast-height shape coefficient, height-diameter ratio and crown-height ratio of the *U. pumila* plantation decreased significantly (*p* < 0.05), with decreases of 8.9, 15.2 and 16.2%, respectively. The ratio of *F. chinensis* increased significantly (*p* < 0.05) by 81.5%. The crown-diameter ratio and stem fullness of the different plantations and the change in the trunk shape of the *S. matsudana* plantations were not significantly affected by the decrease in stand density (*p* > 0.05).

### 3.2 Soil physical characteristics of the forest before and after thinning

#### 3.2.1 Soil bulk density

Table 2 shows the effects of decreased stand density on soil bulk density in the same soil layer at different plantations. There was no significant difference in the soil bulk density changes between the *F. chinensis* and *S. matsudana* plantations (*p* > 0.05). The soil bulk density in the 0–20 cm soil layer at the *U. pumila* plantation decreased significantly (*p* < 0.05), with a decrease of 14.57%.

**Table 2:** Soil physical properties at different plantations before and after thinning.

Physical properties	Soil layer (cm)	Plantations					
		*Fraxinus chinensis*		*Salix matsudana*		*Ulmus pumila*	
		WT	WoT	WT	WoT	WT	WoT
Soil bulk density (g/cm^3^)	0–20	1.48 ± 0.02^Aa^	1.55 ± 0.03^Aa^	1.77 ± 0.04^Aa^	1.77 ± 0.04^Aa^	1.29 ± 0.05^Bb^	1.51 ± 0.02^Aa^
	20–40	1.52 ± 0.02^Aa^	1.56 ± 0.01^Aa^	1.79 ± 0.01^Aa^	1.75 ± 0.07^Aa^	1.57 ± 0.03^Aa^	1.49 ± 0.02^Aa^
	40–60	1.56 ± 0.00^Aa^	1.64 ± 0.02^Aa^	1.75 ± 0.03^Aa^	1.74 ± 0.09^Aa^	1.61 ± 0.02^Aa^	1.60 ± 0.03^Aa^
NCP (%)	0–20	1.8 ± 0.41^Aa^	1.4 ± 0.04^Aa^	1.6 ± 0.25^Aa^	1.9 ± 0.19^Aa^	5.1 ± 1.21^Aa^	1.90.57^Ba^
	20–40	1.8 ± 0.21^Aa^	2.1 ± 0.53^Aa^	1.3 ± 0.18^Aa^	2.8 ± 0.36^Aa^	2.0 ± 0.10^Ab^	1.6 ± 0.64^Aa^
	40–60	1.3 ± 0.39^Aa^	1.5 ± 0.35^Aa^	1.2 ± 0.25^Aa^	2.9 ± 0.52^Aa^	2.5 ± 0.05^Ab^	2.1 ± 0.34^Aa^
CP (%)	0–20	31.1 ± 0.26^Aa^	25.9 ± 1.07^Aa^	3.4 ± 1.50^Aa^	8.0 ± 1.55^Aa^	38.0 ± 1.45^Aa^	30.7 ± 0.71^Ba^
	20–40	27.2 ± 0.66^Aa^	23.1 ± 0.75^Aa^	2.5 ± 1.15^Ba^	8.8 ± 1.64^Aa^	29.0 ± 0.93^Ab^	32.9 ± 2.50^Aa^
	40–60	28.2 ± 0.20^Aa^	24.9 ± 0.92^Aa^	3.4 ± 0.77^Ba^	9.3 ± 2.70^Aa^	30.1 ± 1.28^Ab^	23.7 ± 2.89^Bb^
PT (%)	0–20	32.9 ± 0.57^Aa^	27.3 ± 1.08^Aa^	5.1 ± 1.73^Aa^	9.9 ± 1.68^Aa^	43.1 ± 0.52^Aa^	32.6 ± 1.28^Ba^
	20–40	29.0 ± 0.85^Aa^	25.2 ± 0.31^Aa^	3.8 ± 1.26^Bb^	11.6 ± 1.77^Aa^	30.9 ± 0.83^Ab^	34.5 ± 2.58^Aa^
	40–60	29.6 ± 0.32^Aa^	26.4 ± 0.60^Aa^	4.6 ± 0.59^Ba^	12.2 ± 2.88^Aa^	32.6 ± 1.47^Ab^	25.8 ± 2.55^Bb^
CHC (%)	0–20	21.1 ± 0.37^Aa^	16.8 ± 1.01^Ba^	2.0 ± 0.87^Aa^	4.6 ± 1.00^Aa^	29.5 ± 0.06^Aa^	20.4 ± 0.71^Ba^
	20–40	17.9 ± 0.67^Ab^	14.8 ± 0.51^Aa^	1.4 ± 0.65^Aa^	5.1 ± 1.19^Aa^	18.5 ± 0.65^Ab^	22.0 ± 1.62^Aa^
	40–60	18.1 ± 0.16^Ab^	15.2 ± 0.72^Aa^	1.9 ± 0.42^Aa^	5.6 ± 1.94^Aa^	18.7 ± 0.81^Ab^	14.8 ± 1.83^Ab^
SHC (%)	0–20	22.3 ± 0.60^Aa^	17.7 ± 1.03^Aa^	2.9 ± 1.02^Aa^	5.7 ± 1.11^Aa^	33.6 ± 1.12^Aa^	21.6 ± 1.11^Ba^
	20–40	19.1 ± 0.82^Aa^	16.1 ± 0.30^Aa^	2.1 ± 0.71^Aa^	6.7 ± 1.34^Aa^	19.7 ± 0.60^Ab^	23.1 ± 1.66^Aa^
	40–60	19.0 ± 0.22^Aa^	16.1 ± 0.52^Aa^	2.6 ± 0.32^Aa^	7.2 ± 2.14^Aa^	20.2 ± 0.98^Ab^	16.2 ± 1.62^Ab^

The data are the means ± standard deviations; different lowercase letters in the same column indicate significant differences in different soil layers (*p* < 0.05), and different uppercase letters in the same row indicate significant differences before and after thinning (*p* < 0.05). NCP, noncapillary porosity; CP, capillary porosity; PT, total porosity; CHC, capillary holding capacity; SHC, saturated holding capacity; WoT, without thinning; WT, with thinning.

The effects of stand density reduction on soil bulk density in different soil layers of the same stand revealed that there was no significant difference in soil bulk density between the *F. chinensis* and *S. matsudana* plantations (*p* > 0.05). The soil bulk density in the 0–20 cm soil layer at the *U. pumila* plantation significantly differed (*p* < 0.05), and the soil bulk density of this soil layer was the lowest.

#### 3.2.2 Soil porosity

Table 2 shows the influence of stand density reduction on the soil porosity of the same soil layer at different plantations. There was no significant difference in noncapillary porosity (NCP), capillary porosity (CP) or total porosity (PT) among the *F. chinensis* plantations (*p* > 0.05) before and after thinning. There was also no significant difference in the NCP of the *S. matsudana* plantation (*p* > 0.05), but the soil CP and PT decreased significantly in the 20–40 and 40–60 cm soil layers (*p* < 0.05), with decreases of 72. 0 and 67.2% in the 20–40 cm layer and 63.8 and 62.6% in the 40–60 cm layer, respectively. With respect to the *U. pumila* plantation, the soil NCP increased significantly in the 0–20 cm soil layer (*p* < 0.05), with an increase of 173.3%; the soil CP and PT increased significantly in the 0–20 and 40–60 cm soil layers (*p* < 0.05), with increases of 23.6 and 32.1% in the 0–20 cm layer and 27.2 and 26.22% in the 40–60 cm layer, respectively.

The effects of decreased stand density on soil porosity in different soil layers of the same stand were as follows: the NCP, CP, and PT of the *F. chinensis* plantation did not significantly differ among the soil layers (*p* > 0.05) before and after thinning. There was no significant difference in NCP among the different soil layers or between the initial and thinned density states at the *S. matsudana* plantation (*p* > 0.05); there was a clear reduction in CP and PT in the three soil layers after thinning, and in the WT condition, CP and PT were significantly greater than those in the other two soil layers. The NCP, CP, and PT of the *U. pumila* plantation were significantly different in the 0–20 cm soil layer (*p* < 0.05) than in at least one of the other layers before thinning; the NCP of the soil layer increased after thinning, and the NCP, CP, and PT were greatest under the WT condition.

#### 3.2.3 Soil water holding capacity

Table 2 shows the effects of stand density reduction on the soil water holding capacity (WHC) in the same soil layer of different plantations. The CHC of the *F. chinensis* plantation increased significantly in the 0–20 cm soil layer (*p* < 0.05), by 25.5%; however, there was no significant difference in the soil SHC (*p* > 0.05). There was no significant difference in the soil SHC or CHC of the *S. matsudana* plantation (*p* > 0.05). The SHC and CHC of the *U. pumila* plantation increased significantly in the 0–20 cm soil layer (*p* > 0.05), by 55.0 and 44.7%, respectively.

The influence of decreased stand density on the soil WHC in different soil layers of the same stand was as follows: the CHC of the *F. chinensis* plantation significantly differed in the 0–20 cm soil layer (*p* < 0.05), and the CHC of this soil layer was the greatest; however, there was no significant difference in the soil WHC (*p* > 0.05). There was no significant difference in the SHC or CHC of the *S. matsudana* plantation (*p* > 0.05). The soil SHC and CHC of the *U. pumila* plantation were significantly different in the 0–20 cm soil layer (*p* < 0.05), and both parameters were greater than those in the other layers.

### 3.3 Soil nutrient indices before and after thinning

#### 3.3.1 Soil organic matter

**Figure 1:**
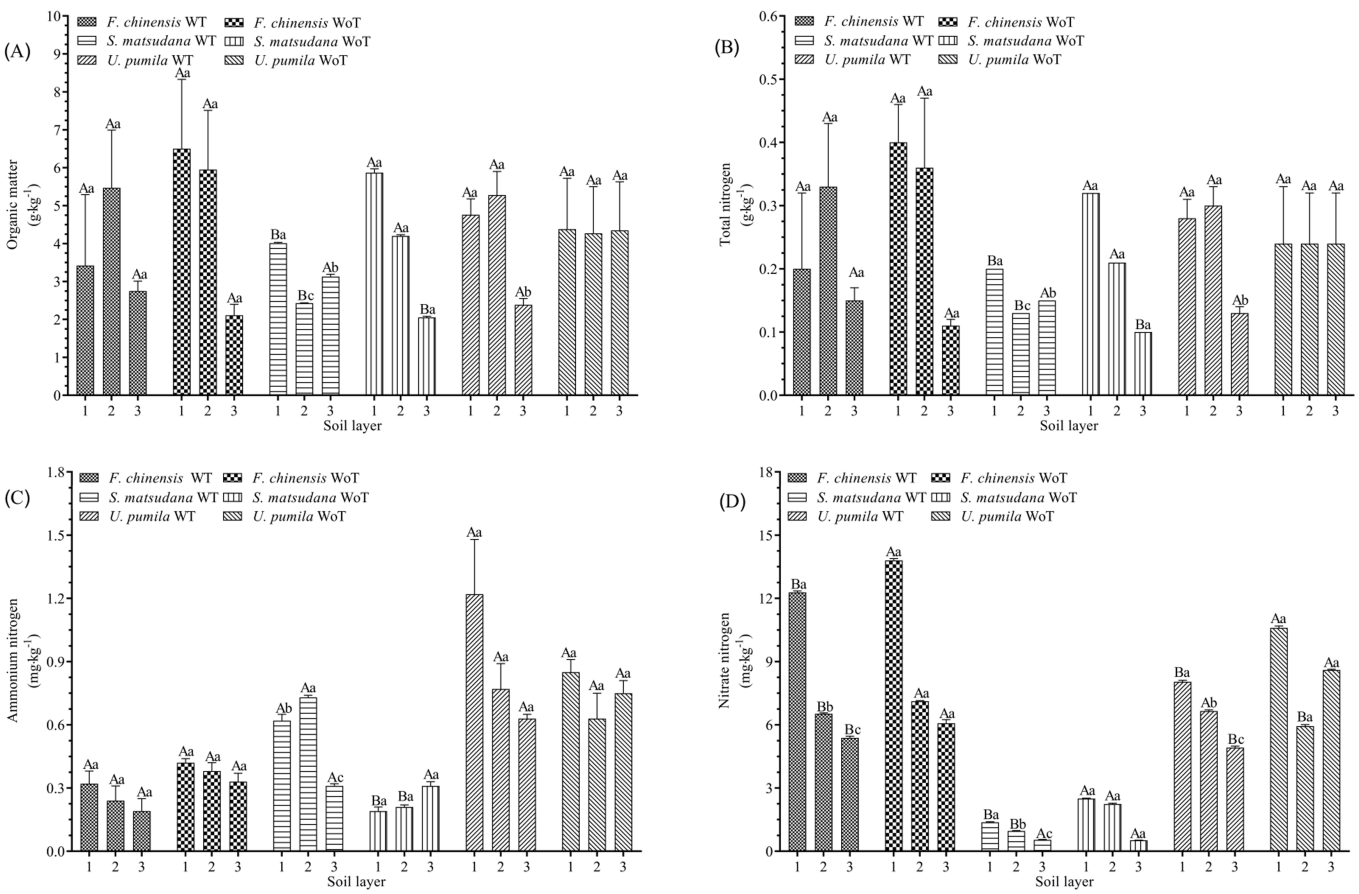
Soil organic matter, total nitrogen, ammonium nitrogen and nitrate nitrogen before and after thinning. **(A)** Soil organic matter before and after thinning. **(B)** Soil total nitrogen before and after thinning. **(C)** Soil ammonium nitrogen before and after thinning. **(D)** Soil nitrate nitrogen before and after thinning. 1: 0–20 cm, 2: 20–40 cm, and 3: 40–60 cm. Different lowercase letters indicate significant differences in different soil layers (*p* < 0.05), and different uppercase letters indicate significant differences before and after thinning (*p* < 0.05). WoT, without thinning; WT, with thinning.

Figure 1A shows the effect of stand density reduction on soil OC in the same soil layer at different plantations. There was no significant difference in soil OC between the *F. chinensis* and *U. pumila* plantations (*p* > 0.05). The soil OC content of the *S. matsudana* plantation decreased significantly in the 0–20 and 20–40 cm soil layers, by 31.7 and 42.1%, respectively, and increased significantly in the 40–60 cm soil layer, by 52.7% (*p* < 0.05).

The influence of stand density reduction on soil OC in different soil layers of the same stand was as follows: there was no significant difference in the soil OC content among the different soil layers of the *F. chinensis* plantation (*p* > 0.05). There were significant differences in soil OC between the different soil layers of the *S. matsudana* plantation (*p* < 0.05), and the value was highest in the 0–20 cm soil layer. The soil OC content of the *U. pumila* plantation significantly differed among the soil layers (*p* < 0.05), and the soil organic matter content was the highest in the 20–40 cm soil layer.

#### 3.3.2 Soil total nitrogen, nitrate, and ammonium nitrogen

Figures 1B–D show the effects of reduced stand density on soil TN, AN, and NN in the same soil layer at different plantations. No significant changes were found in the soil TN or AN in the *F. chinensis* plantation (*p* > 0.05). The soil NN decreased significantly in the 0–20, 20–40, and 40–60 cm soil layers (*p* < 0.05), with decreases of 11.01, 8.3, and 11.4%, respectively. In the *S. matsudana* plantation, the change in soil TN was significant (*p* < 0.05). In addition, the soil TN decreased by 37.5 and 38.1% in the 0–20 and 20–40 cm soil layers, respectively, and increased by 50.0% in the 40–60 cm soil layer. For the same species, the soil NN decreased significantly in the 0–20 and 20–40 cm soil layers (*p* < 0.05), by 37.5 and 38.1%, respectively. Additionally, the soil AN significantly increased in the 0–20 and 20–40 cm soil layers (*p* < 0.05), by 226.32 and 247.6%, respectively. Finally, there was no significant local difference in soil TN or AN at the *U. pumila* plantation (*p* > 0.05), but there was a significant difference in soil NN (*p* < 0.05); in the 0–20 and 40–60 cm soil layers, soil TN and AN decreased by 24.2 and 42.8%, respectively, and the values increased by 12.0% in the 20–40 cm soil layer.

The effects of the decrease in stand density on soil TN, AN, and NN in different soil layers of the same stand were as follows: there was no significant difference in soil TN and AN between the different soil layers at the *F. chinensis* plantation (*p* > 0.05); however, there were significant differences in the soil NN between layers (*p* < 0.05), and this variability was greatest in the 0–20 cm soil layer. At the *S. matsudana* plantation, the soil TN, AN, and NN contents significantly differed among the soil layers (*p* < 0.05), and the soil TN and NN contents in the 0–20 cm soil layer were the highest; the AN content was the highest in the 20–40 cm soil layer. The soil TN significantly differed among the soil layers (*p* < 0.05), and the value was the highest in the 20–40 cm soil layer. There were also significant differences in soil NN among the soil layers (*p* < 0.05), with the greatest difference occurring in the 0–20 cm layer. No significant difference in soil AN was detected among the soil layers (*p* > 0.05).

#### 3.3.3 Soil total phosphorus and available phosphorus

**Figure 2:**
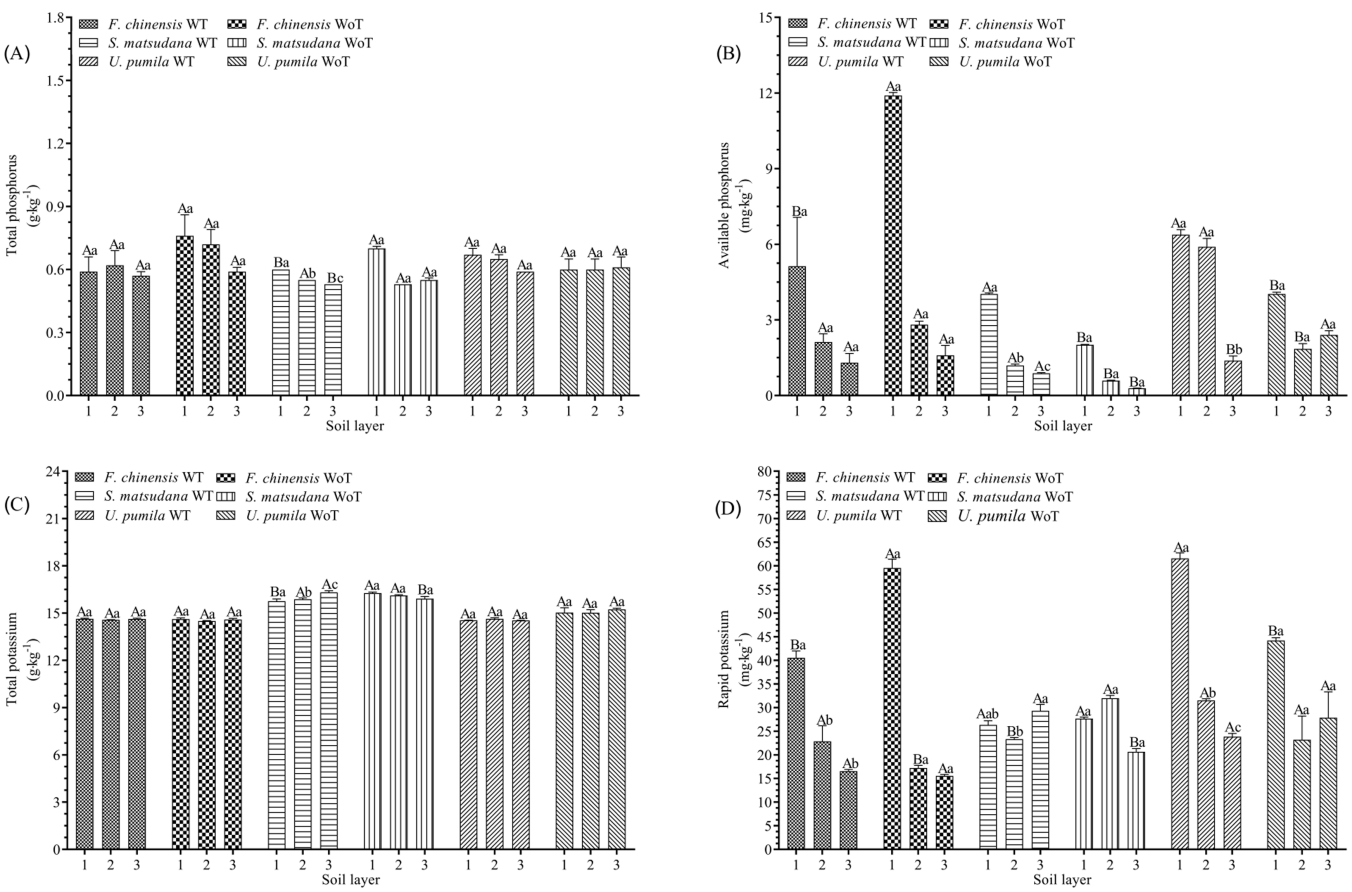
Soil total phosphorus, available phosphorus, total potassium, and available potassium before and after thinning. **(A)** Soil total phosphorus before and after thinning. **(B)** Soil available phosphorus before and after thinning. **(C)** Soil total potassium before and after thinning. **(D)** Soil rapid potassium before and after thinning. Different lowercase letters indicate significant differences in different soil layers (*p* < 0.05), and different uppercase letters indicate significant differences before and after thinning (*p* < 0.05).

Figures 2A,B show that the effects of stand density reduction on soil TP and AP in the same soil layer of different plantations were as follows: there was no significant difference in the soil TP content at the *F. chinensis* plantation (*p* > 0.05), but the soil AP decreased significantly in the 0–20 cm soil layer (*p* < 0.05), by 60.2%. The soil TP at the *S. matsudana* plantation decreased by 14.3 and 3.6% at 0–20 and 40–60 cm, respectively (*p* < 0.05); in contrast, the soil AP increased significantly (*p* < 0.05), with increases of 100.50, 101.7, and 203.5% at 0–20, 20–40, and 40–60 cm, respectively. There was no significant change in the soil TP at the *U. pumila* plantation (*p* > 0.05), but there was a significant difference in the soil AP (*p* < 0.05), since in the 0–20 and 20–40 cm soil layers, the soil TP increased by 58.3 and 218.9%, respectively, whereas in the 40–60 cm soil layer, the soil AP decreased by 42.5%.

The effects of stand density reduction on soil TP and AP in different soil layers of the same stand were as follows: there was no significant difference in soil TP and AP among the different soil layers at the *F. chinensis* plantation (*p* > 0.05). The soil TP and AP at the *S. matsudana* plantation significantly differed among the soil layers (*p* < 0.05), and both variables presented the greatest values in the 0–20 cm soil layer. There was no significant difference in soil TP among the soil layers at the *U. pumila* plantation (*p* > 0.05), but there was a significant difference in the soil AP in the 0–20 cm soil layer.

#### 3.3.4 Soil total potassium and available potassium

Figures 2C,D show that the effects of stand density reduction on soil TK and AK in the same soil layer at different plantations were as follows: there was no significant difference in soil TK at the *F. chinensis* plantation (*p* > 0.05), but there was a significant difference in the soil AK content between the 0–20 and 20–40 cm soil layers (*p* < 0.05). There were significant differences in the soil TK at the *S. matsudana* plantation between the 0–20 and 40–60 cm soil layers (*p* < 0.05). At the *U. pumila* plantation, the soil TK decreased significantly in the 40–60 cm soil layer (*p* < 0.05) and increased significantly in the 0–20 cm soil layer (*p* < 0.05).

The effects of reduced stand density on soil TK and AK in different soil layers of the same stand were as follows: there was no significant difference in soil TK among the soil layers at the *F. chinensis* plantation (*p* > 0.05); however, there was a significant difference in the soil AK in the 0–20 cm soil layer (*p* < 0.05), and the soil AK in this soil layer was the highest. At the *S. matsudana* plantation, the soil TK significantly differed in the 40–60 cm soil layer (*p* < 0.05) and was highest in this layer. The soil AK significantly differed in the 20–40 cm soil layer (*p* < 0.05). There was no significant difference in the soil TK among the soil layers (*p* > 0.05), but there was a significant difference in the soil AK among the different soil layers (*p* < 0.05), and the 0–20 cm soil layer presented the highest concentration.

### 3.4 Correlated changes in dependent variables before and after thinning

**Figure 3:**
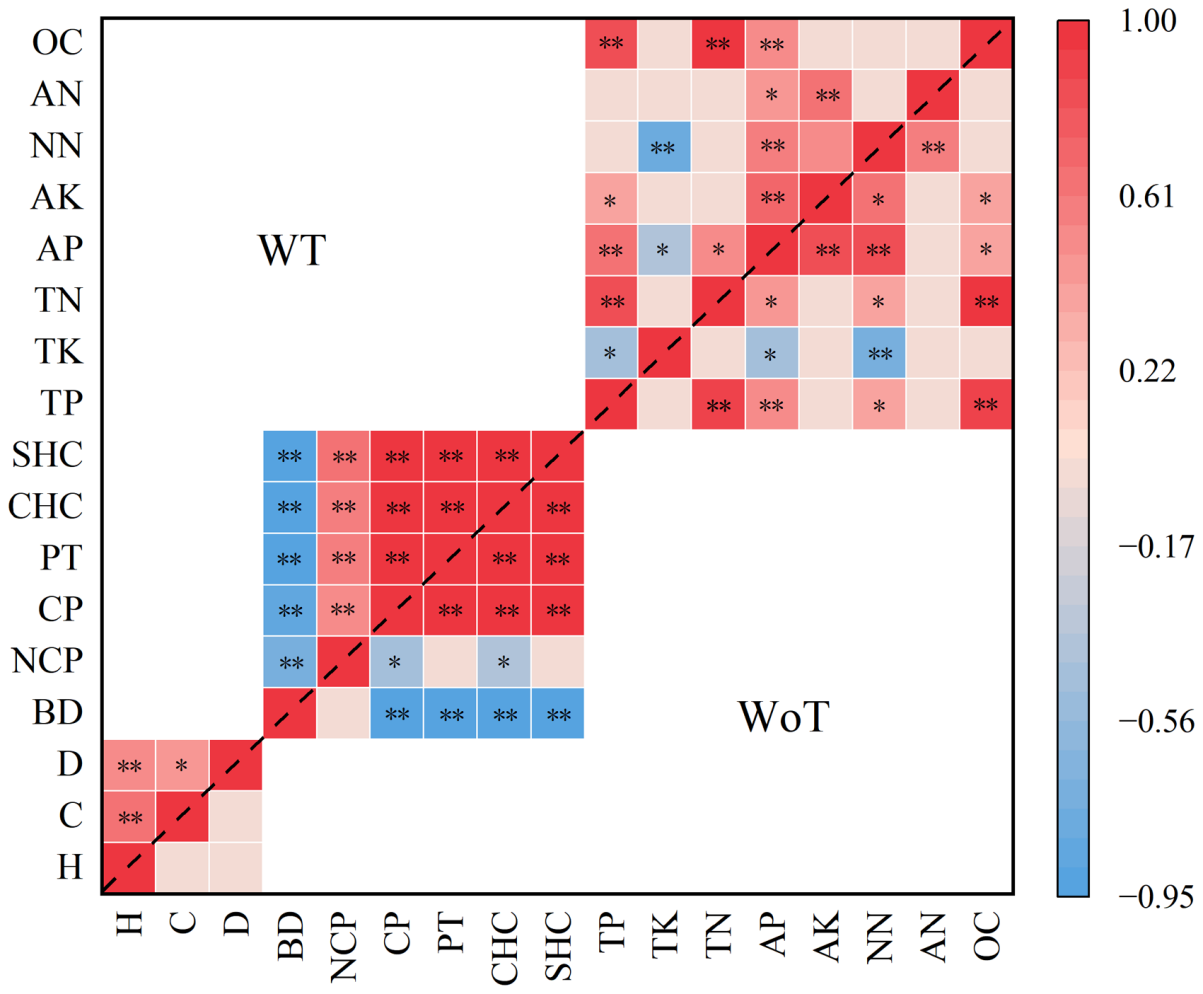
Correlations between various dependent variables before and after thinning of the studied plantations. “**” Represents a highly significant difference (*p* < 0.01). “*” Represents a significant difference (*p* < 0.05). WoT, without thinning; WT, with thinning; H, height; C, crown; D, diameter; NCP, noncapillary porosity; CP, capillary porosity; PT, total porosity; CHC, capillary holding capacity; SHC, saturated holding capacity; TP, total phosphorus; TK, total potassium; TN, total nitrogen; AP, available phosphorus; AK, available potassium; NN, nitrate nitrogen; AN, ammonium nitrogen; OC, organic matter.

Figure 3 shows that the decrease in stand density strongly influenced the correlations between the dependent variables, especially with respect to the different categories. There was a positive correlation between the dependent variables related to stand growth and soil physical properties and a negative correlation among the soil nutrient indices.

The specific impact of thinning on the correlations between different types of dependent variables was as follows: there was a significant positive correlation among crown width, tree height and DBH (*p* < 0.01). The soil bulk density was significantly negatively correlated with NPC, CP, PT, CHC, and SHC (*p* < 0.01). Positive correlations were detected among NCP, CP, PT, CHC, and SHC (*p* < 0.01). The soil bulk density was significantly negatively correlated with NPC, CP, PT, CHC, and SHC (*p* < 0.01). Soil TP was significantly positively correlated with TN, AP, and OC (*p* < 0.01) and significantly negatively correlated with TK after thinning (*p* < 0.05). There was a positive correlation between soil TP and AK (*p* < 0.05). There was a significant negative correlation between soil TK and NN (*p* < 0.01) and a significant negative correlation between soil TK and AP (*p* < 0.05). There was a significant positive correlation between soil TN and AP (*p* < 0.05) and a positive correlation between soil TN and OC (*p* < 0.01). Soil AP was positively correlated with AK, NN, and OC (*p* < 0.01), there was a positive correlation between soil AP and AN (*p* < 0.05), and soil AK was positively correlated with soil NN and AN (*p* < 0.01).

## 4 Discussion

### 4.1 Effect of thinning on the stand growth of artificial plantations

Our results revealed that thinning was conducive to increasing the growth of *U. pumila* plantations. After stand density decreased, there was a significant positive correlation among the dependent variables related to stand growth: crown width, tree height and crown width. The effects of thinning on stem form at the *U. pumila* plantation were mostly reflected in the significant decreases in the DBH, height-diameter ratio and crown-height ratio (*p* < 0.05). Although plants in high-density stands may grow taller due to competition, after thinning, the remaining trees at the *U. pumila* plantation may experience accelerated growth due to enhanced resource acquisition capabilities, particularly in terms of light availability. The significant response of the *U. pumila* plantation compared with the *F. chinensis* plantation and *S. matsudana* plantation after thinning may have resulted from the light-loving characteristics and high sensitivity of this species to thinning measures. In contrast, the impact of thinning on the growth of the *F. chinensis* plantation and *S. matsudana* plantation was not significant, possibly because *F. chinensis* plantation has a slow growth rate and is less competitive for light and space than *U. pumila* is. Although light availability increases after thinning, the deep root distribution of *F. chinensis* allows it to absorb nutrients and water from deep soil layers. Its growth may rely more on nutrients from deep soil layers, but there is no significant change in nutrient and water content in these deep layers before and after thinning. Therefore, the growth-promoting effect of thinning on *F. chinensis* plantations is not significant. Although *S. matsudana* has a faster growth rate than the other species considered, its shallow root system primarily depends on nutrients and water from the topsoil. While light availability increases after thinning, the nutrient content in the topsoil (encompassing organic matter, total nitrogen, ammonium nitrogen, nitrate nitrogen, and total phosphorus) significantly decreases. As a result, the growth of *S. matsudana* may be limited by soil nutrients, and the growth-promoting effect of thinning on *S. matsudana* is not significant. This finding was consistent with most previous research; for example, Bhandari et al. (2021) thinned a redwood (*Bixa orellana*) forest in southwestern Australia and concluded that thinning resulted in greater DBH and tree height, which positively affect diameter and height growth. Sullivan and Sullivan (2016) thinned *Pinus thunbergii* in the Pacific inland area of northwestern North America; their results revealed that the average crown volume of trees is significantly greater than that of unthinned trees at low densities. In another study, Sullivan and Sullivan (2016) conducted thinning treatments on *Pinus thunbergii* plantations in the Pacific inland area of northwestern North America and concluded that trees in medium-to high-density stands tend to have larger diameters (1.7–1.9 times larger than those of unthinned stands) and crowns, but the average height is 5–6 m lower than that of trees in the pine areas of old stands. Notably, thinning optimizes the distance between individual trees and promotes the development of the canopy in the radial direction (Ganbaatar et al., 2021), which results in an increase in tree height.

### 4.2 Effects of thinning on the soil physical properties at artificial plantations

Thinning improved the physical properties of the soil at plantations. In particular, thinning significantly affected the soil physical properties at the *U. pumila* plantation (*p* < 0.05). Thinning reduced the soil bulk density at the *U. pumila* plantation; increased the soil NCP, CP, and PT; increased the CHC and SHC at the *U. pumila* plantation (*p* < 0.05); and affected the soil physical properties of the 0–20 cm soil layer. These findings are consistent with those of most previous studies (Trentini et al., 2020). For example, Kang et al. (2009) reported that *Pinus massoniana* plantations in the Daqingshan area of Guangxi have greater soil water holding capacity and soil porosity and lower soil bulk density-moderate-stand density conditions (1,800 trees hm^−1^) than under other stand density conditions. A study of the effects of thinning on the physical and chemical properties of *Pinus* tree plantations in northwestern Tunisia revealed that thinning improved the physical and chemical properties of the soil compared with those of the soil in a control plot (Jaouadi et al., 2018). Previous studies have shown that the understorey vegetation at *U. pumila* plantations is relatively rich, causing the soil to be relatively loose and increasing soil water storage (Wang et al., 2022). Therefore, changes in factors affecting the surface soil may be attributed to thinning, which reduces stand density and allows the remaining *U. pumila* trees to access more resources (such as light and water), thereby providing their root systems with space for growth and potentially increasing root activity (Cheng et al., 2017; Shu et al., 2021). Additionally, factors such as high litter cover on the surface of *U. pumila* plantations, high microbial activity in the soil, and the high diversity of understory species collectively contribute to disrupting the compact soil structure, increasing the soil porosity, and consequently reducing the soil bulk density while increasing the soil water holding capacity.

### 4.3 Effects of thinning on the soil chemical properties at artificial plantations

The effect of thinning on the TN, TP, and TK contents of the soil was reflected mainly by the significant changes in the soil layers at the *S. matsudana* plantation. Notably, the soil TN, TP, and TK contents decreased significantly in the 0–20 cm soil layer, whereas the changes in the soil nitrogen, phosphorus and potassium contents in the 20–60 cm soil layer slightly differed among the forest types. The results of this study differ from those of Zhao et al. (2023), who reported that the contents of total carbon, TN, TP, TK, AP, and AK were significantly affected by moderate-and high-intensity thinning of a *Picea koraiensis* plantation. The main reason for this discrepancy may be attributed to the larger study area in Zhao’s et al. (2023) research and the inconsistency in thinning density.

Studies have shown that changes in forest structure affect the decomposition rate of litter in forest ecosystems. Large areas of sparseness greatly affect litter decomposition and the contents of nitrogen, phosphorus and potassium in the soil (Comez et al., 2021); thinning can promote soil nitrogen and phosphorus cycling (Zhou et al., 2019); and root nutrient concentrations, relative growth rates and biomass allocation show the greatest plasticity-based responses to nutrient availability (Kramer-Walter and Laughlin, 2017). The soil chemical changes observed in the soil layers of the *F. chinensis*, *S. matsudana*, and *U. pumila* stands were different from those reported in previous studies. The main reasons may be as follows. First, there are differences in species characteristics and root distributions. For example, the roots of *S. matsudana* are distributed mainly in the 40–60 cm soil layer, affecting nutrient availability and soil chemistry in this layer. In contrast, most of the roots of *U. pumila* are distributed in the 0–40 cm soil layer, influencing the WHC and nutrient content of the upper soil. In other studies, the root distribution of species such as Brazil nut trees or larch may be relatively deep, leading to differences in nutrient absorption efficiency and soil layer impacts (de Oliveira et al., 2024). Second, differences in thinning intensity and stand management practices exist. The intensity and management of thinning operations can significantly affect the amount of solar radiation reaching the forest floor, soil water cycling, and nutrient transformation (Wang T. et al., 2021). For example, in studies on *F. chinensis*, *S. matsudana*, and *U. pumila*, specific density adjustments were applied (e.g., from 3 m × 3 m to 3 m × 6 m). After thinning, the increased surface solar radiation in the *S. matsudana* stands accelerated soil nutrient cycling, leading to decreases in the TN, TP, and TK contents in the 0–20 cm soil layer. In contrast, the thinning intensities reported in other studies may have been relatively high, resulting in different impacts on soil chemical properties (Li X. et al., 2023; Zhao et al., 2023). Third, seasonal differences in soil nutrient dynamics. For example, in the studies of *F. chinensis*, *S. matsudana*, and *U. pumila*, soil samples may have been collected during seasons with relatively stable nutrient levels, whereas studies of Brazil nut trees involved sampling during seasons with active nutrient cycling (e.g., the rainy season). Additionally, in Mediterranean forest ecosystems in the Cuenca Mountains (southeastern Spain), biochemical, microbial, and physicochemical variables are influenced by treatment and seasonal factors (summer and autumn) after thinning and restoration (Baena et al., 2013). Fourth, differences in soil types and climatic conditions exist. For example, the study areas of *F. chinensis*, *S. matsudana*, and *U. pumila* may differ from those of other studies in terms of soil types (e.g., sandy soil vs. clay) and climatic conditions (e.g., temperate vs. tropical), leading to variations in the effects of thinning on soil chemical properties (Jian et al., 2024). These factors collectively contribute to significant differences in the impacts of thinning on soil chemical properties across different tree species and stands.

### 4.4 Effects of thinning on stand growth and soil physical and chemical correlations at artificial plantations

Thinning had a more significant effect on the correlations among stand growth indices, soil physical indices and soil nutrient indices than on the relationship among the other indices. The effect of thinning on the relationship between stand growth index, soil physical index and soil nutrient index was greater than that on the relationships among the other indices. Thinning increased the correlations among DBH, crown width and tree height during stand growth, which was consistent with the results of Gao et al. (2022), Li M. et al. (2023), and Li X. et al. (2023). This may be because thinning reduces stand density, reduces resource competition (especially light), and increases the growth space of the remaining trees. The correlation between soil physical and chemical properties caused by thinning tended to increase, which was consistent with the results of Wang et al. (2011), Zhou et al. (2018), and Li X. et al. (2023). This may be related to the decrease in soil bulk density after thinning, the increases in soil porosity and soil water holding capacity, and the root growth of vegetation. This study focused on the short-term effects of thinning on the growth and soil improvement of typical stands in the Yellow River Delta. However, to obtain a more reliable long-term forest management strategy for the effects of thinning on stand growth and soil improvement, future studies should focus on long-term continuous observations of soil physical and chemical properties, underground microbial community composition and soil root growth dynamics. The short-term effects after thinning can provide important basic data and theoretical support for subsequent research and provide a scientific basis for optimizing the intensity and frequency of thinning.

## 5 Conclusion

Thinning affects the growth index and physical and chemical properties of artificial plantation soil increases the correlation among DBH, crown width and tree height at plantations and increases the correlation between soil physical and chemical properties, depending on the species. Thinning promoted the growth of *U. pumila* specifically, DBH, tree height and crown width increased significantly but did not significantly promote the growth of *S. matsudana* or *F. chinensis* plantation, which provides a scientific basis for optimizing forestry management strategies. In addition, thinning plays an important role in soil improvement and soil and water conservation by improving soil physical properties, such as reducing soil bulk density and increasing soil porosity and water holding capacity, especially in areas with severe soil erosion. Understanding the effect of thinning on the soil nutrient content is helpful for ecological restoration and biodiversity improvement. In particular, significant changes in the nitrogen, phosphorus, and potassium content in the *S. matsudana* plantations may play a significant role in these processes. In this work, the positive effects of thinning on plantation growth and soil physical and chemical properties in the Yellow River Delta, are revealed, and specific a practical findings for improving forestry management and ecological restoration in the region are introduced. The experimental data can also be used to develop and verify forest growth and soil change models to predict the long-term effects of different thinning strategies.

## Data Availability

The original contributions presented in the study are included in the article/supplementary material, further inquiries can be directed to the corresponding authors.
